# An integrative mating system assessment of a nonmodel, economically important Pacific rockfish (*Sebastes melanops*) reveals nonterritorial polygamy and conservation implications for a large species flock

**DOI:** 10.1002/ece3.3579

**Published:** 2017-12-03

**Authors:** Kurt W. Karageorge, Raymond R. Wilson

**Affiliations:** ^1^ Department of Biological Sciences California State University Long Beach CA USA

**Keywords:** genetic incompatibility, polyandrogyny, polyandrous females, polygynandry, premating, sexual selection, Viviparity‐Driven Conflict Hypothesis

## Abstract

Characterizing the mating systems of long‐lived, economically important Pacific rockfishes comprising the viviparous *Sebastes* species flock is crucial for their conservation. However, direct assignment of mating success to sires is precluded by open, offshore populations and high female fecundity. We addressed this challenge by integrating paternity‐assigned mating success of females with the adult sex ratio (ASR) of the population, male evolutionary responses to receptive females, and reproductive life history traits—in the framework of sexual selection theory—to assess the mating system of *Sebastes melanops*. Microsatellite parentage analysis of 17 pregnant females, 1,256 of their progeny, and 106 adults from the population yielded one to four sires per brood, a mean of two sires, and a female mate frequency distribution with a truncated normal (random) pattern. The 11 multiple paternity broods all contained higher median allele richness than the six single paternity broods (Wilcoxon test: *W* = 0, *p* < .001), despite similar levels of average heterozygosity. By sampling sperm and alleles from different males, polyandrous females gain opportunities to enhance their sperm supply and to lower the cost of mating with genetically incompatible males through reproductive compensation. A mean of two mates per mated female with a variance of one, an ASR = 1.2 females per male, and the expected population mean of 2.4 mates for mated males (and the estimated 35 unavailable sires), fits polygamous male mate frequency distributions that distinguish polygynandry and polyandrogyny mating systems, that is, variations of polygamy, but not polyandry. Inference for polygamy is consistent with weak premating sexual selection on males, expected in mid‐water, schooling *S. melanops*, owing to polyandrous mating, moderately aggregated receptive females, an even ASR, and no territories and nests used for reproduction. Each of these characteristics facilitates more mating males and erodes conspicuous sexual dimorphism. Evaluation of male evolutionary responses of demersal congeners that express reproductively territorial behavior revealed they have more potential mechanisms for producing premating sexual selection, greater variation in reproductive success, and a reduced breeding effective population size of adults and annual effective size of a cohort, compared to *S. melanops* modeled with two mates per adult. Such divergence in behavior and mating system by territorial species may differentially lower their per capita birth rates, subsequent population growth, and slow their recovery from exploitation.

## 1 INTRODUCTION

### 1.1 The importance of understanding mating systems

Mating systems fundamentally impact the ecology, evolution, and extinction of populations, and require our scientific inquiry for conservation. A mating system embodies the relationships among reproductively receptive individuals, their offspring, and the environment over the reproductive season (Emlen & Oring, 1977), and can generate sexual selection from competition for fertilization, that occurs directly between members of the same sex (e.g., male–male competition and sperm competition), or indirectly, between the attraction of one sex to the other (e.g., female choice; Andersson, 1994; Darwin, 1871). Premating competition mechanisms create variation in mating success among individuals that, in combination with any postmating competition, results in variation in their reproductive success and sexual selection (Bateman, 1948; Jones, Arguello, & Arnold, 2002; Wade, 1979; Warner, Shapiro, Marcanato, & Petersen, 1995), principally when more adults of one sex do not mate (sensu Darwin, 1871; Shuster, 2009). Consequential to conservation, mating systems with strong directional sexual selection and reproductive exclusion, and/or with sexual conflict (different reproductive interests of males and females), experience an associated reduction in effective population size, *N*_e_ (Holland & Rice, 1999; Nunney, 1993; Parker & Waite, 1997; Warner et al., 1995). Reproductive behavior also affects many biological processes that differentially impact *N*_e_ (Anthony & Blumstein, 2000; Baer & Schmid‐Hempel, 1999; Darden & Croft, 2008; Hoglund, 1996), including inbreeding (Meagher, Penn, & Potts, 2000) and offspring viability (Newcomer, Zeh, & Zeh, 1999). Ultimately, mating systems and sexual selection may promote the evolution of populations through reproductive isolation and speciation (reviewed in Panhuis, Butlin, Zuk, & Tregenza, 2001), or increase their extinction risk (Møller, 2000; Møller & Legendre, 2001). Accordingly, the assessment of a species’ mating system is a vital step for conservation (Parker & Waite, 1997; Vincent & Sadovy, 1998) and is the purpose of this study.

### 1.2 Mating systems and the conservation of commercially exploited fish

The severe reduction in many commercially exploited marine fish populations has resulted in little to no increase in their postcollapse abundance, despite over two decades of potential recovery with declining fishing mortality (Hutchings & Reynolds, 2004). At low population sizes, the per capita population growth rate, *r*, may decline due to demographic stochasticity and positive density dependence (i.e., Allee effects; Møller & Legendre, 2001), and disrupt normal mating system function. Delays in the optimal timing of reproduction, a reduced intensity of social interactions, skewed sex ratios, and reduced/skewed mating and fertilization success can lower birth rates and *N*_*e*_ below an already low census size (Anthony & Blumstein, 2000; Parker & Waite, 1997; Rowe & Hutchings, 2003). Moreover, intense sexual selection within some mating systems can weaken the recovery of small populations (Blumstein, 1998; Møller, 2000; Møller & Legendre, 2001).

Pacific rockfishes of the viviparous genus *Sebastes* are a unique component of biodiversity because they encompass the criteria that define a species flock (Johns & Avise, 1998). Explosive speciation of the genus *Sebastes* has generated over 100 species that have adapted throughout most of their endemic range. *Sebastes* species occur in pelagic and demersal environments along the continental shelf, slope, and seamounts of the North Pacific Ocean—in contrast to sister genera (e.g., *Hozukius*,* Sebastiscus*, etc.; Kendall, 2000). Yet, populations of many rockfish species are overfished remnants of their former large sizes, while we lack a mating system assessment for any of the estimated 70 *Sebastes* species in the northeastern Pacific. A barrier to mating system studies in nature arises from the difficulty of obtaining the means and variances of mating and reproductive success for a breeding population, especially for nonmodel marine organisms, like Pacific rockfishes. Large, open populations coupled with high fecundity prohibits direct assignment of mating success to the nonpregnant sex (or the nonbrood guarding sex), that is, unavailable parents—mated males/sires for *Sebastes* species—because a candidate pool of sires cannot be identified (Jones & Ardren, 2003). Fortunately, integrating paternity estimates of female mating success with the population constraint of the adult sex ratio, and the evolutionary responses of males to receptive females in the framework of sexual selection theory—can help diagnose mating systems of *Sebastes* species.

Since Darwin's (1871) formulation of sexual selection theory, it has been evident that the expression of secondary sex traits depends on the mating system (including parental roles), and that strong reciprocal relationships between mating systems and their strength and direction of sexual selection exist (Andersson, 1994; Arnold, 1994; Bateman, 1948; Jones & Avise, 2001). Darwin (1871) described species with intense polygyny (males with highly variable mating success, relative to monandrous females) possessing “strongly marked” sexual differences, but less so in moderately polygynous animals, and little dimorphism in monogamous species. Contemporary evidence for sexual dimorphism as an evolutionary response to the intensity of premating sexual selection comes from microsatellite‐based studies of genetic mating systems. Polyandry, referred to in this study as a population‐level mating system where females are variable in their mating success, whereas each male has at most one mate, was discovered in the strongly sexually dimorphic pipefish *Syngnathus scovelli* (Jones, Walker, & Avise, 2001). Slight, transient sexual dimorphism is expressed by *S. typhle*, where polygynandry, a form of polygamy, was appraised (Jones, Rosenqvist, Berglund, & Avise, 1999). An intriguing feature of *Sebastes* species is the striking, inconspicuous sexual dimorphism between the sexes (Lenarz & Wyllie Echeverria, 1991; Love, Morris, McCrae, & Collins, 1990; Wyllie Echeverria, 1986), which lack sexually selected secondary sex characters such as male weaponry, strong sexual size dimorphism, body patterning, and male nuptial colorations. Also absent are secondary sex traits associated with the environment that are not used in rockfish reproduction: nests for all *Sebastes* species (because they are viviparous), and courtship/mating territories by the mid‐water, schooling species that are reproductively nonterritorial. *In situ* observations convey that the most prominent male secondary sex trait is a transient, behavioral visual signal: courtship displays, either in the water column, or on territories (Helvey, 1982; Shinomiya & Ezaki, 1991). We posit that if multiple paternity is documented in a *Sebastes* species (Van Doornik, Parker, Millard, Berntson, & Moran, 2008; Hyde et al., 2008; Sogard et al., 2008), with schooling, nonterritorial behavior as adults, a likely genetic mating system would be a form of polygamy, as monogamy and polygyny are disqualified. Polyandry is also possible, but *Sebastes* species do not show sex‐role reversal characteristics in behavior or morphology (reviewed in Gwynne, 1991): females do not appear to compete for males (Helvey, 1982; Shinomiya & Ezaki, 1991), and no elaborated secondary sex traits are evident among females.

**Figure 1:**
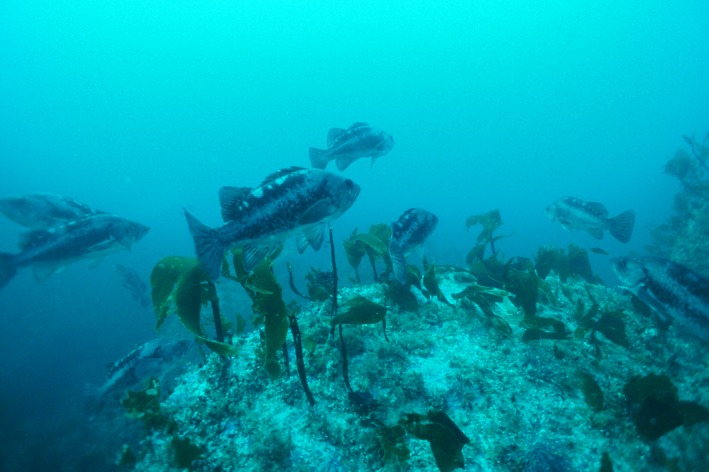
A school of adult *Sebastes melanops*, over a shallow water temperate rocky reef near the Redfish Rocks Marine Protected Area off the coast of southern Oregon, USA. Photo courtesy of Oregon Department of Fish and Wildlife

We assess the mating system of *S. melanops* (Figure 1), using an integrative framework. Adults in the breeding population and offspring from pregnant females are used to estimate the mating success of mothers and indirectly estimate the average mating success of the unavailable mated males. These mating system measures are then used to evaluate a male mate frequency distribution corresponding to a (expected) null mating system hypothesis of polyandry, against the alternative hypothesis of polygamy. Inference on the mating system of *S. melanops* is also supported by comparing the evolutionary responses of males to receptive females between *Sebastes* species comprising two major life history groups and by assessing the expected strength of premating sexual selection on males. We also examine the effect of female mating strategies on the genetic diversity of broods and their implications for female fitness. Lastly, we model the effect of the supported *S. melanops* mating system on the relationship between annual effective population size and reproductive failure and show how divergence of behavior and mating system of congeners with an alternate life history could alter this relationship.

## 2 MATERIALS AND METHODS

### 2.1 Sample collection

We sampled the recreational fishery landings for *S. melanops* adults caught at nearshore rocky reefs (<30 m) off Newport, Oregon, U.S.A. During January through March (2004), the season of *S. melanops* fertilization and embryonic development (Bobko & Berkeley, 2004), we surveyed adults for gravid females and females with whole‐brood absorption of ova, which could indicate the occurrence of unmated adult females. Due to the high fecundity of female broods, the number of offspring per brood was randomly sampled, and the minimum desired sample size was chosen to detect sires that might fertilize a small fraction of ova. Binomial power analysis indicated that 58 offspring per brood were needed to detect at least one paternal allele/offspring from a father that sired only 5% of a brood, with a 0.95 probability (sensu Bartley, Kent, & Drawbridge, 1995; see Sokal & Rohlf, 1995 for binomial power analysis). To account for some offspring that would fail to amplify, for example, due to small tissue samples, degraded DNA, and Chelex resin occasionally in the PCR, we sampled 96 offspring per brood to fill 96‐well DNA extraction plates.

During September through November (2007), the mating season of *S. melanops* (Wyllie Echeverria, 1987), we sampled adults from the population to estimate the adult sex ratio, so that the population demographic inference on the average number of mates of mated males could be drawn. We also collected DNA tissue from adults to assess their genetic variation and to calculate the combined exclusionary probability of various locus combinations to discriminate a random male in the population as being the sire of a brood (Jones, 2005). *Sebastes melanops* that measured at least 360 mm total length, the length at 50% population maturity for males (which experience a smaller size at maturity than females; Wyllie Echeverria, 1987), were further examined for sex and maturity based on the external morphology of ovaries and testes (Gunderson, Callahan, & Goiney, 1980). Binomial power analysis indicated that 74 adult *S. melanops* were needed for a 0.95 probability of encountering rare alleles with a relative frequency ≥0.02 in the adult population (Bartley et al., 1995; Sokal & Rohlf, 1995). Maternal fin clips, whole ovaries with offspring, and adult fin clips were isolated and preserved in the field with 95% ethanol and then stored at 4°C.

### 2.2 Preparentage analysis: DNA extraction, PCR, genotyping, locus performance and population genetic analysis with selected loci

We tested congeneric microsatellite loci on the adult population sample to assess their amplification quality, level of polymorphism, allele frequencies, heterozygosities, and agreement of genotype frequencies to Hardy–Weinberg proportions (HWP) for parentage analysis. Genomic DNA was extracted using a standard 5% Chelex digestion (Wilson & Donaldson, 1998), and loci were amplified in each 6‐μl PCR that included a final concentration of 1× *Taq* buffer, 1–2 mmol/L MgCl_2_, 0.07–0.10 μmol/L of each forward and reverse primer, 0.2 mmol/L of each DNTP, 0.1 U *Taq* polymerase, and 25–100 ng of template DNA. PCR conditions for *S. melanops* were as described by Miller, Schulze, and Withler (2000) for *Sal1* and *Sal3* loci, developed from Pacific Ocean perch, *S. alutus* (GenBank Accession numbers AF153595 and AF153597, respectively); and by Gomez‐Uchida, Hoffman, Ardren, and Banks (2003) for the *Spi6* locus, developed from canary rockfish, *S. pinniger* (GenBank no. AY192600). However, we optimized annealing temperatures at 46°C for *Sal1* and 58°C for *Sal3*. PCR products were resolved on a LI‐COR 4300 DNA Analyzer and scored with the Saga 2 Microsatellite Analysis Program (LI‐COR, Lincoln, Nebraska, U.S.A.). We selected *Sal1*,* Sal3*, and *Spi6* loci to genotype *S. melanops* mothers and their progeny. These loci displayed little nonspecific amplification relative to other polymorphic loci we tested on the adult population sample, yielded 11 to 18 alleles per locus, and produced a multilocus exclusion probability of 0.977. Genotypes were analyzed with ARLEQUIN version 3.5 (Excoffier & Lischer, 2010). The genotype frequencies of the adult population sample did not deviate from HWP (*Sal1*:* H*_o_ = 0.86, *H*_e_ = 0.90, *p *=* *0.59; *Sal3*:* H*_o_ = 0.90, *H*_e_ = 0.85, *p *=* *0.30; *Spi6*:* H*_o_ = 0.84, *H*_e_ = 0.82, *p *=* *0.17), nor show significant linkage disequilibrium between loci (*Sal1*/*Sal3*,* p *=* *0.06; *Sal1*/*Spi6*,* p *=* *0.11; *Sal3*/*Spi6*,* p *=* *.06). Additional steps taken to minimize scoring error included amplifying all samples at least twice (in separate PCR sessions) to compare scoring consistency between replicates. We also verified the inheritance of maternal alleles in progeny against the genotype of their known mother and tested maternal allele frequencies in each progeny array for departures from an expected 1:1 Mendelian segregation ratio.

### 2.3 Paternity analysis and inference on female mate numbers

Paternity analysis of each *S. melanops* brood was accomplished by reconstructing the minimum number of sires that could explain the three‐locus progeny array sample with known mother, using the computer program GERUD2.0 (Jones, 2005). When more than one combination of paternal genotypes for a given number of sires was reconstructed, then the most likely paternal genotypic combination was accepted. We estimated the relative paternity share for each sire within all multiple sired broods, based on the number of progeny assigned to each sire (i.e., reconstructed paternal genotype), but we tested for significant paternity skew only in broods with one paternal solution; that is, genotypic combination of sires. We used GERUDsim2.0 (Jones, 2005) to test the accuracy of GERUD2.0 to correctly determine the true number of sires of each progeny array, employing the number of offspring assigned to each sire and the adult population allele frequencies.

We estimated the harmonic mean number of mates of mated females, which equals the average of the inverse of the number of mates per female, from the paternity results over all 17 females. It represents the fraction of an average female's brood that an average male is expected to sire (i.e., population average paternity share per male), and indicates the possible intensity of sperm competition among mated males, under a model of sperm mixing and equal paternity by all inseminating males, when females mate at least once, and each male mates with a particular female only once (Shuster & Wade, 2003; Wade & Arnold, 1980).

### 2.4 Effect of female mating strategy on the genetic diversity of broods

We compared the genetic diversity, as average observed heterozygosity and median allele richness, between broods that were sired by multiple males and broods that were sired by a single male. We also tested for a significant difference in the observed heterozygosity between all pooled offspring and the adult population sample. Statistical tests and graphics were produced with the statistical program R (R Core Development Team, 2016; http:/www.r-project.org).

### 2.5 Population demographic inference on the average mate frequency of the unavailable parents: mated males

Predicting the average number of mates for mated males in a population is possible, even when paternity assignment of progeny and all mates to individual males is not possible, because the total number of mates that bear progeny with females must equal the total number of mates of males (Fisher, 1958). Consequently, the average number of mates per male and female is joined through the sex ratio (Arnold & Duvall, 1994; Shuster & Wade, 2003), that we estimated as the ratio of the number of adult females to the number of adult males: *ASR* = *n*_mature ♀_/*n*_mature ♂_. We estimated the average number of mates of mated females in the breeding population, ${\overset{¯}{x}}_{f}^{*}$ (and the variance, $v_{f}^{*}$), from the number of reconstructed sires of brooded females, so that the average number of mates per mated male, a key mating system parameter, could be estimated with the equation: ${\overset{¯}{x}}_{m}^{*}=\left(\mathrm{ASR}\right){\overset{¯}{x}}_{f}^{*}$ (Shuster & Wade, 2003). The parameter estimates draw inference on *mated individuals* and do not include unmated adults. We assume that the unknown proportions of unmated adult males and females within the ASR are similar (i.e., similar sizes of the zero mate frequency classes). Potential violations of this assumption and biases of parameter estimates are also evaluated. The expected strength of premating sexual selection on males, an indicator of the magnitude of unmated males, is addressed below. To assess which male mate frequency distributions and corresponding (genetic) mating systems were consistent with *S. melanops*, we compared the observed mean and variance of the number of mates of mated females (${\overset{¯}{x}}_{f}^{*},\;v_{f}^{*}$), the *ASR*, and the expected average number of mates of mated males (${\overset{¯}{x}}_{m}^{*}$), against the means and variances of male mate frequency distributions that characterize the following mating systems. Polyandry (*H*_o_), when strong premating directional sexual selection on females is likely (and strong sexual dimorphism is elaborated in females), resulting in females that are much more variable in their mating success than males, producing a much larger zero mate frequency class of unmated females than the zero class of unmated males (if present). Polyandrogyny (*H*_a_), when weak directional sexual selection on females is expected (and weak sexual dimorphism may be inconspicuous), resulting in females that are somewhat more variable in their mating success than males, producing a larger zero class of unmated females than the zero class of unmated males. Polygynandry (*H*_a_), when weak directional sexual selection on males is expected (and weak sexual dimorphism may be inconspicuous), resulting in males that are somewhat more variable in their mating success (and a larger zero class of unmated males than the zero class of unmated females; Shuster & Wade, 2003).

### 2.6 Evaluation of the evolutionary responses of males to female spatiotemporal distributions in receptivity for *S. melanops* and other congeners in the large marine species flock

To further assess the mating system of *S. melanops* and predict other possible mating systems of *Sebastes* species for conservation, we followed the mating system classification framework of Shuster and Wade (2003), in combination with a comparative approach to identify suites of behaviors and characters that could produce—or be the result of—different sexual selection mechanisms, and hence mating systems, and that could facilitate appropriate management plans (Harvey & Pagel, 1991; Vincent & Sadovy, 1998) for this large species flock. Emlen and Oring (1977) showed that the spatiotemporal distributions of receptive females affects the distribution of mating success among males, yet the distribution of paternity is modified by the ability of males to (1) engage in sperm competition, (2) search for or guard females and/or territories, (3) provide care of offspring, (4) cooperate with or exploit their mates (i.e., sexual conflict), or to (5) change mating strategy (Shuster & Wade, 2003). We evaluated these evolutionary responses of males to female receptivity for *S. melanops*, by integrating our mating system parameter estimates with previous observations (elucidated by a comprehensive literature search) on the reproductive behavior and biology of *S. melanops* and congeners with a similar adult life history and ecology, characteristic of conspecific schooling behavior as adults, occurrence in mid‐water above substrata (Love et al., 1990; “pelagic state”, Kendall, 2000), and are confirmed and/or presumed to be reproductively nonterritorial. We then evaluated male evolutionary responses separately for congeners that exhibit solitary behavior or form loose aggregations as adults, typically occur on or near the substrata (Love et al., 1990; “demersal state”, Kendall, 2000), and are confirmed to be reproductively territorial (Shinomiya & Ezaki, 1991), or expected to be (Gingras, Ventresca, Donnellan, & Fisher, 1998) due to strong territorial behavior for feeding and shelter areas (Larson, 1980a,b), and long‐term site fidelity (R. Larson, pers. comm.). Differences in the evolutionary responses of males between the two life history groups were compared in the context of mating system and sexual selection theory, for their potential influences on shaping the mating system and associated effective population size.

### 2.7 The influence of male and female reproductive life history and ecology on shaping the sex difference in opportunity for selection

We also evaluated the underlying influences of male and female reproductive life history and ecology on shaping the expected sex difference in opportunity for selection, or opportunity for sexual selection, which indicates the overall expected strength of premating directional sexual selection on males of *S. melanops* and congeners with a schooling, nonterritorial life history (and also for congeners with a demersal, territorial life history). These evaluations provide theoretical support for some of the observed and expected/apparent types of evolutionary responses of males to female receptivity, and inference on the mating system (Appendix S1).

### 2.8 The influence of the mating system on the relationship between annual effective population size and reproductive failure

We modeled the influence of different mating systems for their predicted effects on the relationship between the annual effective population size, *N*_e_ (i.e., within‐season and nonoverlapping generations, or a cohort), and the proportion of breeding adults/whole broods that experience reproductive failure, modifying the approach of Parker and Waite (1997). We applied different demographic variables and reproductive failure assumptions to Wright's (1931) equation *N*_e_ = (4*n*_m_*n_f_)/(*n*_m_ + *n*_f_), where *n*_m_ and *n*_f_ are the number of breeding males and females. We defined the breeding sex ratio (*BSR*) as the ratio of adults that successfully mate, relative to the initial adult population size of *N* = 100 adults, with 50 adults per sex (= *ASR* =1); the number of mates per individual; and the effects of reproductive failure on *N*_e_ of a cohort, for each mating system, as follows. For polygamy (polyandrogyny and polygynandry), the *BSR* = *ASR* = 1; no adults were excluded from mating. The number of mates per adult is 2, the paternity share of all broods = 0.5, and for each female's unsuccessful brood (e.g., due to male sterility or postzygotic brood failure), *n*_f_ is reduced by one, whereas that sire's progeny/alleles are still represented in another female's brood; therefore, *n*_m_ is reduced by one for every two females that experience reproductive failure. For monogamy, the *BSR* = *ASR* = 1 and for every brood failure, all of a mating pair's progeny are eliminated from the cohort of the next generation. For moderate polygyny, the BSR = 1♂:2♀, where 50% of the adult males are excluded from mating by sexual selection, and the other 50% of males have two mates each, completely siring each of their mates’ broods. The progeny of one male are eliminated from the cohort for every two brood failures. Polyandry was modeled in a similar manner: 50% of adult females have no mates (BSR = 2♂:1♀) and 50% mate with two males each. Here the relative paternity share of all broods = 0.5, and every reproductive failure eliminates progeny from three parents (each male has one mate). Greater premating sexual selection was modeled by extreme polygyny, where only 10% of the males mate, each with 10% of the females, to the exclusion of other males.

## 3 RESULTS

### 3.1 Paternity analysis and inference on female mate numbers

**Figure 2:**
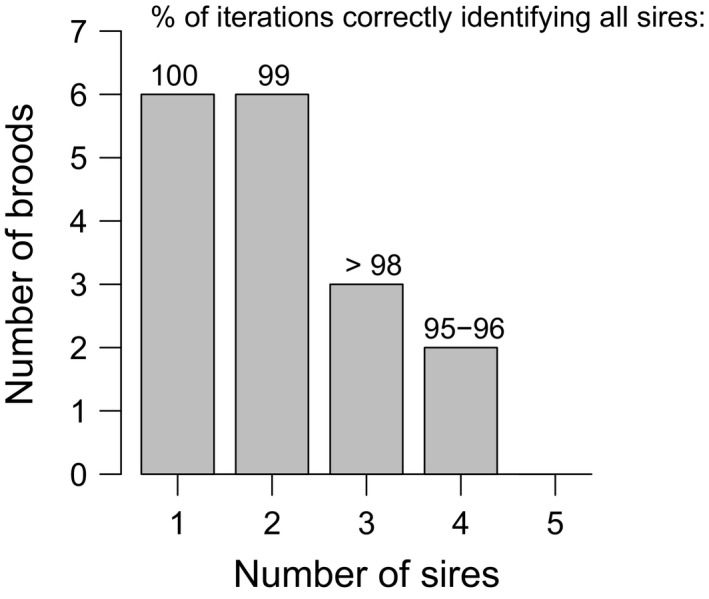
Microsatellite parentage analysis results of 17 field‐collected broods of *Sebastes melanops*. Female multiple mating is common in this population, with 65% of the broods sired by two or more males. Above each bar (mate frequency class) is the percent of simulation iterations that matched the number of reconstructed sires determined by the parentage program, using the number of offspring per sire per brood and the population allele frequencies

Paternity analysis of the 17 brooding mothers revealed that a minimum of 52 parents (17 mothers and an estimated 35 sires) produced the 1,256 genotyped offspring. The effective number of males siring a brood varied from one to four sires, and 65% of the broods were sired by two or more males, the population frequency of multiple paternity (Figure 2). Simulations on each progeny array indicated that we correctly identified the true number of sires fertilizing each brood with a minimum probability of 0.95, that was lowest for the two broods sired by four males (Figure 2; see Table S1 for specific broods). Maternal alleles were inherited by progeny in the expected 1:1 Mendelian ratio for equal segregation, in 49 of the 51 locus‐by‐brood groups (i.e., 3 loci × 17 broods = 51 groups/tests; Table S1). The probability of two or fewer “false positives” of 51 tests is 0.52.

**Figure 3:**
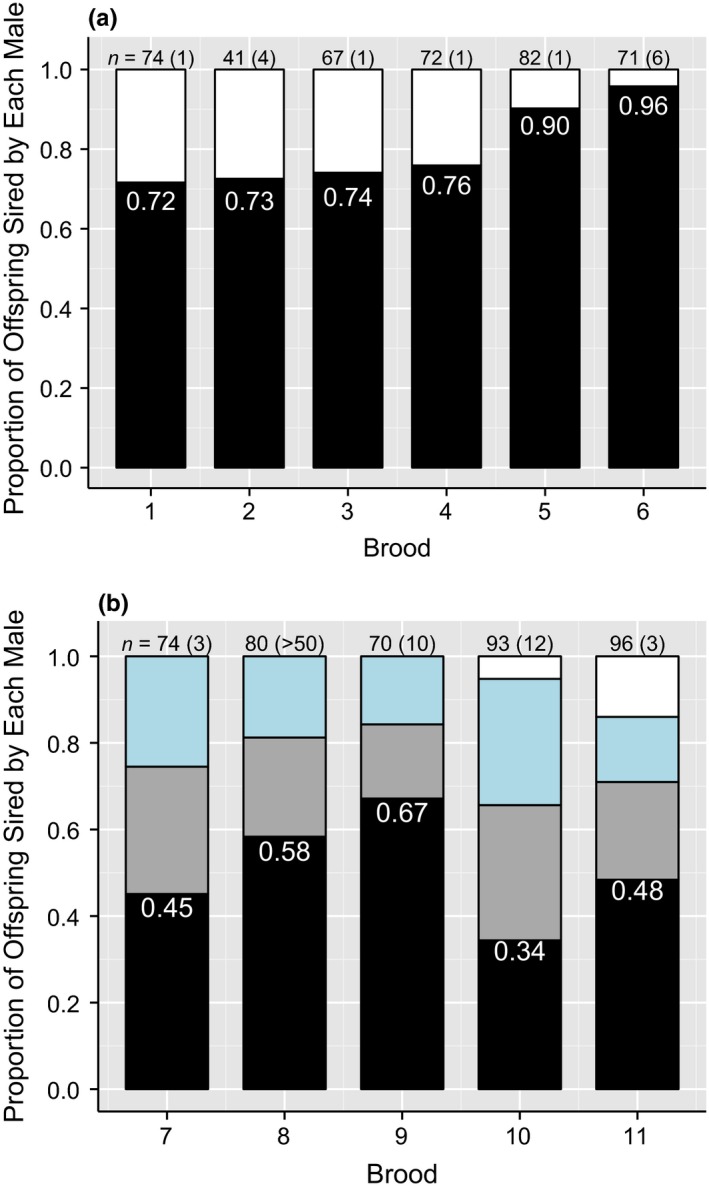
Relative paternity share of sires within multiple sired broods (i.e., females with two, three, or four subclutches, each with a different sire). The estimated proportion of offspring sired by the male with the largest paternity share for each brood is indicated within each bar. Above the bars are the number of offspring genotyped, followed by the number of possible paternal genotypic solutions for the number of sires detected, in parentheses. (a) relative paternity share within broods sired by two males; (b) relative paternity share within broods sired by three and four males (showing the most likely relative paternity share)

Skewed paternity was unambiguously revealed in four broods, where two sires and one paternal genotype solution were reconstructed. In each of these broods, one male sired a significant majority of the sampled progeny (*H*_o_ = equal paternity between males, chi‐square tests: *χ* ^2^ > 13.8, *df* = 1, *p *<* *0.001 for each of the four broods; Figure 3a). The most extreme case of skewed paternity occurred in a brood where 68 of the 71 genotyped progeny (96%) most likely had the same father. In broods sired by three and four males, the relative paternity share was more equitable among most of the sires, based on the most likely paternal genotype solution (Figure 3b). Yet the relative paternity share of these broods were less certain, due to more than one paternal genotype solution for each brood. This resulted in different proportions of progeny assigned to the three or four reconstructed sires of those broods. The estimated harmonic mean number of mates of mated females in the population was 0.62.

### 3.2 Effect of female mating strategy on the genetic diversity of broods

**Figure 4:**
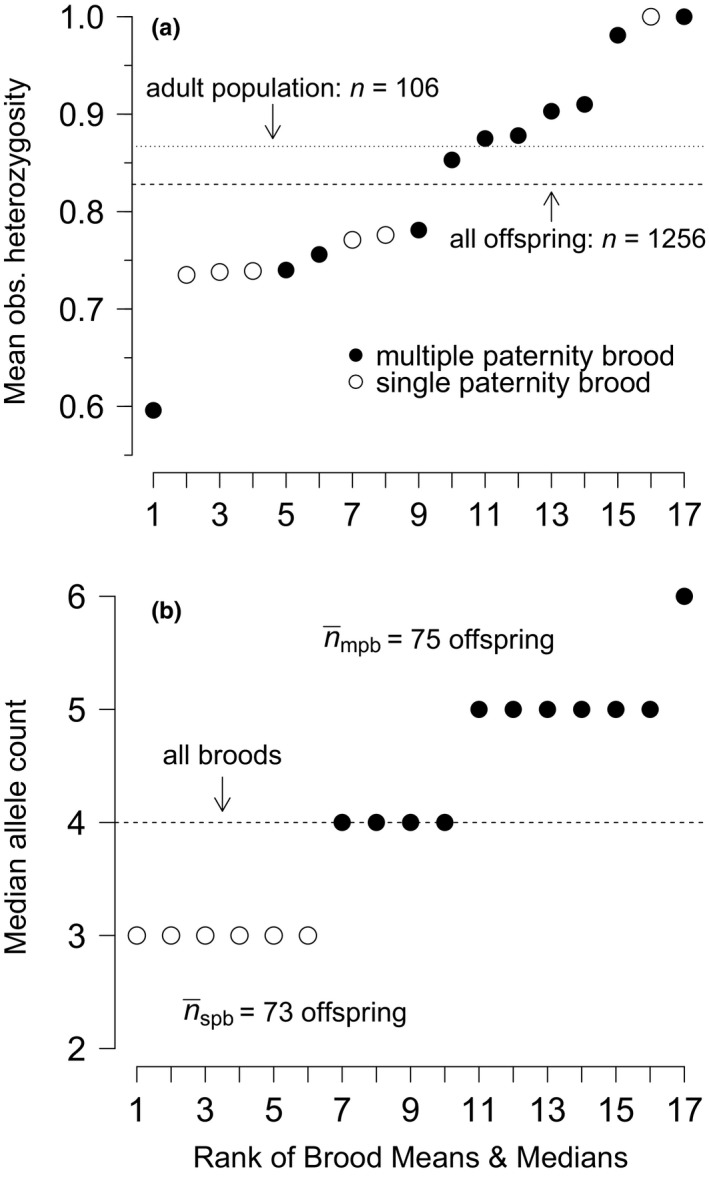
Effect of female mating strategy on the genetic diversity of broods: monandrous mating females were revealed from (effectively) single paternity broods (circles); polyandrous mating females were revealed from multiple paternity broods (dots). (a) The sampling distributions of mean observed heterozygosity for single and multiple paternity broods, ranked from lowest to highest. The difference between the two cumulative probability distributions (not shown) was not significant, due to substantial overlap of variation in brood heterozygosities. (b) The sampling distributions of median allele richness for single and multiple paternity broods ranked from lowest to highest. Multiple sired broods ranked significantly higher in median allele richness than did single sired broods

Polyandrous mating did not have a statistically significant effect on the average observed heterozygosity of multiple sired broods, relative to monandrous mating and (effectively) single sired broods: the cumulative probability distributions of average observed heterozygosity of multiple paternity broods (820 progeny from 11 broods) and single paternity broods (432 progeny from six broods) were similar (Kolmogorov–Smirnov two‐sample test statistic = 31, *p *>* *0.10; Figure 4a). However, multiple paternity broods were greater in median allele richness than single paternity broods (Wilcoxon test: *W* = 0, *p *<* *0.001; Figure 4b). The total offspring sample from multiple paternity broods carried an average of about three more distinct alleles per locus than the total offspring sample from single paternity broods. The observed heterozygosity between all pooled offspring and the adult population sample was not statistically different at any locus (*Sal1*: χ^2^ = 0.05, *df* = 1, *p *>* *0.82; *Sal3*: χ^2^ = 2.30, *df* = 1, *p *>* *0.13; *Spi*6: χ^2^ = 0.81, *df* = 1, *p *=* *0.37).

### 3.3 Population demographic inference on the average mate frequency of mated males

**Figure 5:**
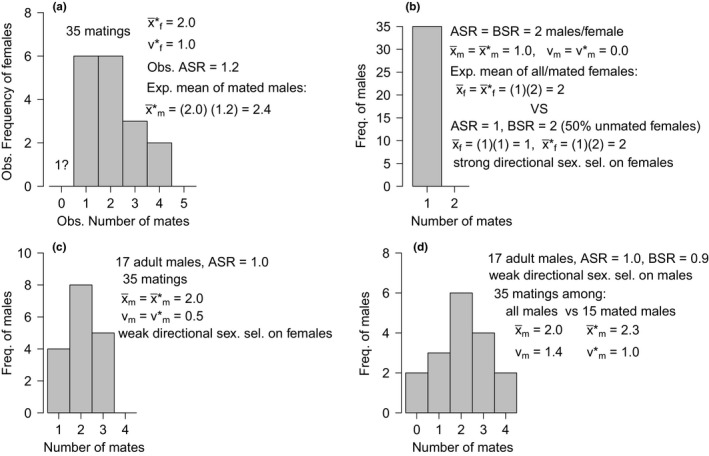
Population demographic inference on the average number of mates of the unavailable parents, mated males (${\overset{¯}{x}}_{m}^{*}$), based on the mean and variance of the observed mate frequency distribution of adult females (${\overset{¯}{x}}_{f}^{*},\;v_{f}^{*}$), and the observed adult sex ratio, ASR (a). The expected ${\overset{¯}{x}}_{m}^{*}$ compared to the means and variances of male mate frequency distributions characteristic of (b) polyandry, where all males mate at most once, (c) polyandrogyny, where both sexes are variable in their mating success, but more so among females, and (d) polygynandry, where both sexes are variable in their mating success, but more so among males. The observed ${\overset{¯}{x}}_{f}^{*},\;v_{f}^{*}$, ASR, and the expected ${\overset{¯}{x}}_{m}^{*}$ are inconsistent with polyandry hypothesized under two different conditions (when the expected average number of mates per mated female matches that observed from the data), but are in agreement with polygamous males and polyandrogyny and polygynandry mating systems, or variations of polygamy

The mean number of mates of the gestating females was ${\overset{¯}{x}}_{f}^{*}=2.0$, with variance $v_{f}^{*}=1.0$. Our survey of hundreds of adult females for unfertilized, atrophied broods, which could be the result of mating failure, yielded only one potential case. The adult sex ratio sample was *ASR* = 1.2, yielding a predicted mean number of mates per mated male of ${\overset{¯}{x}}_{m}^{*}=\left(2.0\right)\left(1.2\right)=2.4$. The observed mean and variance of mated females, the *ASR*, and the predicted ${\overset{¯}{x}}_{m}^{*}$ (Figure 5a) were inconsistent with a mean and variance of males with a mate frequency distribution characteristic of polyandry (Figure 5b), where two conditional outcomes are possible when the expected average number of mates of mated females matched that observed from gravid females (i.e., ${\overset{¯}{x}}_{f}^{*}=2$). Our observations and predicted ${\overset{¯}{x}}_{m}^{*}$ were consistent with a mean and variance of a male mate frequency distribution exhibiting polyandrogyny (Figure 5c), and consistent with a mean and variance of a male mate frequency distribution characteristic of polygynandry (Figure 5d). We evaluated a potentially upward‐biased ${\overset{¯}{x}}_{m}^{*}$ from possible sampling error in the *ASR*, due to underestimating adult males and/or unmated males, which would decrease the *total* average male mating success of mated and unmated males, ${\overset{¯}{x}}_{m}$, to less than the mated male average, ${\overset{¯}{x}}_{m}^{*}=2.4$ (Fig. S1).

### 3.4 Evaluation of the evolutionary responses of males to female spatiotemporal distributions in receptivity for *S. melanops* and congeners

In addition to population inference for polygamy among mated males, weak conspicuous sexual dimorphism, limited alternate mating strategies/phenotypes (due in part to female control of copulation), and other evolutionary responses of males to female spatiotemporal distributions in receptivity were consistent with moderate levels of male mating success and weak directional sexual selection on males. These support a nonterritorial polygamy mating system classification for *S. melanops* (Table 1; Appendix S1). However, several reciprocal evolutionary responses in the reproductive behavior, phenotypes, and ecology between *Sebastes* species that exhibit schooling, nonterritorial behavior (NT) as adults, compared to congeners that express occasional solitary, and demersal, territorial behavior (TT) as adults, were evident (Table 2). Among observed NT *Sebastes* species, males search for females that are in small groups, usually consisting of multiple males surrounded by a single female (i.e., OSR = 1 to 7♂'s per ♀), in mid‐water, sometimes near conspecific schools, and males are noticeably smaller than females. In contrast, among observed TT species, females with transient dichromatism search for solitary territorial or lekking males, on the bottom, and males are noticeably larger.

**Table 1:** Evaluation of the evolutionary responses of males to female spatiotemporal distributions in receptivity for *Sebastes melanops* and congeners that express schooling, nonterritorial behavior, and the supported mating system classification of *S. melanops*

Observed male–female courtship and mating associations	*S. melanops*: pair copulated twice in lower half of water column (October)[Link]; *S. emphaeus*: 1 of 2 ♂'s (ca. 10 cm) appeared to copulate ♀ (ca. 15 cm) several times, while slowly ascending from ca. 20 m to 5 m of water surface, near large conspecific school (August)[Link]; *S. mystinus*:** ♂'s search for** ♀**'s in mid‐water groups** of 1 to 7 ♂'s per ♀[Link]
Alternate mating strategies	Sperm competitor: remating same ♀'s[Link] and possible variable sperm allocation in response to ♀ size[Link] ^,^ [Link]; sneaky copulations by ♂'s unlikely with small urogenital papilla and required ♀ cooperation for mating: slow swimming speed and ventral mounting by ♂'s in water column[Link] ^,^ [Link] ^,^ [Link]; alternate mating phenotypes constrained by small expected sex difference in opportunity for selection, ∆I[Link] (see Table S3)
Sperm competition	Possible, average paternity share of an ave. *S. melanops* brood is 62% with sperm mixing and equal paternity,[Link] apparent in broods sired by ≥3 ♂'s (Figure 3b); remating partners[Link] ^,^ [Link] may increase ave. paternity share, reduce **♀** mating freq.; pattern of testes recrudescence in *Sebastes* indicates sperm still available after one or more matings[Link]; postmating sexual selection if some ♂'s disproportionately fertilize most/all of their mates’ ova[Link]
Male parental care	None, ♂ energy and time devoted to **searching for** ♀'s,[Link] courtship persistence and displays,[Link] ^,^ [Link] mating with multiple ♀'s[Link] &/or remating partner(s)[Link]
Potential for sexual conflict	High, premating SC over mating frequency and size‐specific fecundity,[Link] enhanced by polygamy[Link]; with viviparity, possible postmating intra‐ and intergenomic conflicts b/w: parental genomes within embryos, sibling embryos, and mothers and embryos can generate genetic incompatibility and postzygotic isolation[Link] ^,^ [Link]
Sexual dimorphism	Weak, except for elaborate ♂ courtship displays[Link] ^,^ [Link]; **♂**'s **smaller than** ♀'s[Link] ^,^ [Link] ^,^ [Link] a possible response to ♀ search costs[Link] and courtship persistence; urine from enlarged ♂ urinary bladders[Link] may allow ♀'s to assess ♂ reprod. status[Link]; sex. dim. eroded by small expected ∆I[Link]
Supported mating system	Nonterritorial polygamy; exp. ave. no. of mates of mated males ${\overset{¯}{x}}_{m}^{*}=2.4$,[Link] total mean ♂ mating success less with unmated adult ♂'s[Link]; population estimates and observations consistent with predicted variance in ♂ mating success for polyandrogyny or polygynandry, not effectively zero variance for polyandry (Figure 5)

Sources:

1. Following precopulatory swimming behaviors and fin‐flaring by ♂'s, copulation was described as ventral contact between partners and body quivering by ♂'s, while ♀'s slowly swim (Wayne Palsson, pers. comm., NOAA Fisheries); 2. Helvey (1982); 3. Shapiro, Marconato, and Yoshikawa (1994); 4. Marconato and Shapiro (1996); 5. Shinomiya and Ezaki (1991); 6. Shuster and Wade (2003); 7. Wyllie Echeverria (1987); 8. Shuster, Briggs, and Dennis (2013); 9. Chapman, Arnqvist, Bangham, and Rowe (2003); 10. Holland and Rice (1999); 11. Zeh and Zeh (2001); 12. Zeh and Zeh (2000); 13. Lenarz and Wyllie Echeverria (1991); 14. Stacey, Kyle, and Liley (1986); 15. Shuster (2009).

Notes: Text in bold indicates a reciprocal difference between the reproductive behavior, phenotype, or ecology of *Sebastes* species that are demersal, often solitary as adults, and express reproductively territorial behavior.

Estimated in this study.

**Table 2:** Evaluation of the evolutionary responses of males to female spatiotemporal distributions in receptivity for *Sebastes* species that are benthic/demersal, and express reproductively territorial behavior, and their hypothesized mating system classifications

Observed male–female courtship and mating associations	*S. miniatus*: ♀ courted after entering presumed territory of solitary ♂, 1 ♂ per ♀ during courtship sequences (1 conspecific within 4 m of one pair), <2 m off bottom, ♂'s larger than ♀'s[Link]; *S. inermis*:** ♀'s search for territorial ♂'s**, ♂ courtship rejection, multiple mating by ♂'s and ♀'s, remating, and sperm leakage after complex mating sequence[Link]
Alternate mating strategies	As with reprod. nonterritorial spp. (Table 1); ♀'s above bottom required for ventral mounting and internal fertilization by ♂'s
Sperm competition	Likely in *S. inermis* [Link]; large/old experienced territorial ♂'s could attain higher insemination success due to complex courtship/mating patterns, higher sperm production, and inseminate most/all of their mates’ reproductive tracts until sperm limited
Male parental care	None, ♂ energy and time devoted to **securing/defending mating territories** [Link] ^,^ [Link], courtship displays, mating with multiple ♀'s &/or remating partner(s)[Link]
Potential for sexual conflict	Yes, premating SC potentially greater than in reprod. nonterritorial spp. due to unique premating sexual selection mechanisms: SC over mating[Link] due to ♀ preference for quality of ♂ territory (e.g., size) or position effect enhancing ♀ encounter rate[Link] (e.g., highest territory on a rocky reef), and opportunities for ♀'s to copy the mate choice of other ♀'s (imitative behavior)[Link]; postmating SC with viviparity (see Table 1)
Sexual dimorphism	Weak, except for ♂ courtship displays and **territorial behavior** [Link] ^,^ [Link]; **larger ♂'s in territorial spp** [Link] ^,^ [Link] ^,^ [Link] favors acquiring mating territories; urine from enlarged ♂ urinary bladders[Link] may allow ♀'s to assess ♂ reprod. status[Link]; **lighter ♀'s in ** ***S. miniatus*** and ***S. inermis*** **during courtship** aids in conspecific sex ID: ♀'s avoid territorial ♂ aggression[Link]; sex. dim. eroded by polyandrous mating and high variation in female fecundity[Link]
Hypothesized mating systems	Territorial polygamy (strong polygynandry), convergence to polygyny possible in some species with greater sexual selection on males; **territorial ** ***S. inermis*** **exhibit major attributes of lekking species** [Link] ^,^ [Link]

Sources:

1. Gingras et al. (1998); 2. Shinomiya and Ezaki (1991); 3. Warner et al. (1995); 4. Genner et al. (2008); 5. Dugatkin and Godin (1993); 6. see also Lenarz and Wyllie Echeverria (1991); 7. Stacey et al. (1986); 8. Noble (1938); 9. Shuster and Wade (2003); 10. Wyllie Echeverria (1987); 11. Hoglund and Alatalo (1995).

Note: Text in bold indicates a reciprocal difference between the reproductive behavior, phenotype, or ecology of *Sebastes* species that express mid‐water/schooling behavior as adults, and reproductively nonterritorial behavior.

### 3.5 The influence of the mating system on the relationship between effective population size and reproductive failure

**Figure 6:**
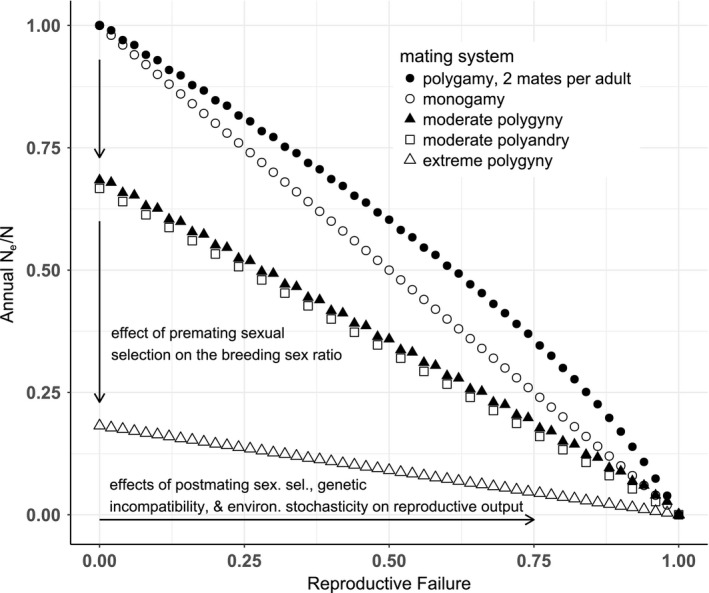
The influence of the major types of mating systems on the relationship between the ratio of the annual effective population size, *N*_e_ (Wright, 1931) to the adult population size, *N* = 100 (sex ratio equal), and the proportion of reproductive failures due to postmating processes (male sterility, genetic incompatibility, and natural selection). Nonterritorial polygamy in *Sebastes melanops*, modeled with two mates per adult (filled circles), is expected to yield higher effective sizes to offspring of a cohort, compared to reproductively territorial *Sebastes* species with the same adult population size (moderate polygyny, open squares; extreme polygyny, open triangles), due to less opportunities for sexual selection on males. Sexual selection acting on females also reduces N_e_ with polyandry (filled triangles), relative to polygamy and monogamy (open circles). See text for the breeding sex ratios, the average number of mates per breeder, and the relative paternity share of broods for each mating system (modified from Parker & Waite, 1997)

Our modeled rate of within‐season *N*_e_ decline in response to reproductive failure of breeding adults was faster for polygamy, modeled after *S. melanops* with two mates per adult, and for moderate polygyny, than those two mating systems modeled by Parker and Waite (1997). Relative to other mating systems, polygamy still conferred the highest *N*_e_ to a cohort for a fixed adult population size and a given level of reproductive failure, due to weak premating sexual selection and offspring/alleles of each mating male represented in two female broods (Figure 6).

## 4 DISCUSSION

### 4.1 Female mate frequencies, patterns of paternity within broods, and inference for polyandrous females and a random mating pattern

As a result of multiple male inseminations in 11 of the 17 gravid females from this natural population of *Sebastes melanops*, polyandrous mothers gain opportunities for direct (material) and indirect (genetic) benefits to enhance their reproductive success and offspring viability, relative to monandrous mothers (Evans & Magurran, 2000; Newcomer et al., 1999). These females were sired from one to at least four males, yielding a mate frequency distribution resembling a truncated normal distribution, which could occur do to random mating. A larger sample of adult females could have increased the accuracy of the estimated distribution, and the probability of detecting less common females in the population with five mates, if present. Some females may have also failed to mate (and would represent a zero mate frequency class), and/or experience reproductive failure; we found one mature female with an atrophied brood (discussed below). Van Doornik (2008) also documented broods sired by one to four males in *S. alutus*, although only 20 offspring per female were genotyped. However, the probability of underestimating the true number of sires per brood increases considerably when competing sires fertilize a small fraction of ova, as we found (see also Sogard et al., 2008), requiring larger offspring samples per brood (Jones, 2005; Neff & Pitcher, 2002). Our inference on detecting the true number of sires for each *S. melanops* brood was achieved with high probability, based on our sampling protocols that utilized power analysis, and simulations testing the likelihood of observed paternity outcomes.

What major factors drive the number of mates per female to vary in a random fashion? In terms of direct benefits, this may partly depend on the quantity of semen acquired per mate (*sensu* mate fecundity; Arnold & Duvall, 1994), that can covary with male differences in size/age, recent mating history, and their possible differential allocation of sperm (i.e., remating same female or not; Wedell, Gage, & Parker, 2002). Importantly, the reproductive biology and few observations of rockfish mating behavior indicate mating is demanding, involving ventral mounting by males in the water column while in motion, and a small male urogenital papillae relative to body size, for internal fertilization (Table 1); we note the sperm leakage of mating *Sebastes inermis* seen by Shinomiya and Ezaki (1991). Female traits, including size/age and fecundity, also influence the number of mates they choose (Jones, Rosenqvist, Berglund, & Avise, 2000; Trexler, Travis, & Dinep, 1997). Thus, multiple mates and remating with the same partner (seen with *S. melanops*, Wayne Palsson, pers. comm., NOAA Fisheries), may be important for females to obtain an adequate sperm and seminal fluid supply, especially for prolonged sperm storage in some *Sebastes* species (Takahashi, Takano, & Takemura, 1991).

The cause(s) of the significantly skewed paternity shares we found in all broods with two sires will remain elusive without unfeasible experiments that control for mating order and sperm quantity in this extremely fecund, long‐lived viviparous species. Skewed paternity may simply reflect variation in ejaculate quantities between sperm competing males, as noted previously, due to sperm mixing and paternity proportional to the number of sperm inseminated per male (Wade & Arnold, 1980; Wedell et al., 2002). These females may have mated with a second male due to an inadequate sperm quantity from their first mate. Sogard et al. (2008) also found skewed paternity between two sires in several *S. atrovirens* broods. Other explanations for biased paternity include sperm precedence depending on mating order (Zeh & Zeh, 1994), cryptic female choice (Eberhard, 1996), and when the sperm from different sires vary in their genetic compatibility with a female's ova (Zeh & Zeh, 1996; discussed below). Our females that were effectively sired by three and four males showed more even paternity shares that would be expected with sperm mixing and paternity proportional to (compatible) sperm number per male, and when there are no mating order effects biasing paternity with several sires (Zeh & Zeh, 1994).

### 4.2 Effect of female mating strategy on the genetic diversity of broods and possible evolutionary repercussions for the viviparous *Sebastes* species flock

Other potential benefits from multiple male inseminations important to females, consistent with their random pattern of mate frequencies, are more apparent from our results that show the effect of female reproductive behavior on the genetic diversity of their progeny. Polyandrous mating did not increase average heterozygosity of multiple sired broods, relative to (effectively) monandrous females with single sired broods, despite that nearly twice the number of broods/progeny analyzed were from polyandrous females. This result is in agreement with life history theory, which posits that polyandrous mating is not expected to be an adaptive response to bet‐hedging for additive genetic variance in large populations with high genetic diversity (Yasui, 1998), like those for *S. melanops*. Genetic diversity/*N*_e_ is also maintained by large brood sizes (sensu Waples, 1987), and particularly by the mating system (Parker & Waite, 1997; Fig. 6, discussed below). Yet our discovery that allele richness was higher in all multiple sired broods suggests that if a small fraction of these progeny survive and disperse into new marine environments, they will retain more of the genetic composition of the parent population when establishing small founder populations, which experience faster declines in allele richness than in heterozygosity (Luikart, Aitken, & Allendorf, 2013).

Although we did not measure differences in reproductive success (RS) nor monitor the survival of progeny from monandrous and polyandrous females, higher allele richness in all multiple sired broods exposes a link between the potential benefits of polyandrous mating and female RS. Genetic incompatibility between the gametes of mates, such as when an incompatible allele is in a specific genotype of their zygotes, can produce nonadditive genetic variation (as with overdominance and epistasis), with negative impacts on offspring viability and individual RS (Newcomer et al., 1999; Olsson & Madsen, 2001; Zeh & Zeh, 1996). Bearing the hallmark signal of genetic incompatibility, zygote/embryo failure, Boehlert, Barss, and Lamberson (1982) found that the percent of expected fecundity decreased with increasing embryonic developmental stage in *S. entomelas*, where eyed embryo fecundity was only 38% of the expected weight‐specific fecundity. Length‐specific fecundity estimates revealed a 25% decline in larvae from ova numbers in *S. schlegeli* (Boehlert, Kusakari, & Shimizu, 1986), and a similar decline was evident between ova and zygote stages in *S. melanops* (Bobko & Berkeley, 2004). By sampling sperm (and alleles) from different males, polyandrous females reduce the risk of mating with an infertile male, and gain opportunities to exploit sperm competition and/or female choice in their reproductive tract to assess oocyte–sperm genotype compatibility (Olsson & Madsen, 2001; Zeh & Zeh, 1997). Additionally, because *Sebastes* species are live‐bearing, the postzygotic reallocation of maternal resources from defective to viable offspring (reproductive compensation), provides a final defense to minimize the cost and/or risk of fertilization by genetically incompatible sperm (Zeh & Zeh, 1997, 2001). Fetomaternal conflict and intragenomic conflict specific to viviparity could contribute to postzygotic genetic incompatibility and favor the maintenance of polyandrous mating (Zeh & Zeh, 2001) in *Sebastes* species. Polygamy, inferred in this study, can also intensify sexual conflict relative to monogamy, and increase the evolutionary potential for reproductive isolation (Holland & Rice, 1999; Rice, 1998). Remarkably, despite pelagic dispersal of larvae, long distance migrating adults of many species, and species overlap (with multispecies aggregating schools), hybrids are mysteriously uncommon within the *Sebastes* species flock (for examples of exceptions, see Seeb, 1998; Muto, Kai, Noda, & Nakabo, 2013), and allopatric speciation is clearly insufficient to explain *Sebastes* diversification. However, weak premating directional sexual selection and polyandrous females, genetic incompatibility, and less frequent viable hybrids among viviparous species pairs, compared to oviparous species pairs, presumably due to postzygotic barriers to viable interspecific hybridization (and/or hybrid sterility), are also the central components of the Viviparity‐Driven Conflict Hypothesis for sympatric speciation (Zeh & Zeh, 2000).

### 4.3 Evolutionary responses of males to female spatiotemporal distributions in receptivity and inference on the mating system of *Sebastes melanops*

Our evaluation of male evolutionary responses to female spatiotemporal distributions in receptivity for schooling, nonterritorial *Sebastes* species revealed evidence supporting male polygamy and a nonterritorial polygamy mating system classification for *S. melanops*. On a fine spatiotemporal scale, we estimated that mated *S. melanops* males mate with 2.4 females on average, though not from reconstruction of sires with matching multilocus genotypes between broods—an extremely unlikely outcome given this large, open breeding population. Alternatively, we integrated paternity results of the broods with the population constraint of the *ASR* to estimate the average mating frequency of mated males (${\overset{¯}{x}}_{m}^{*}$), because the mean number of mates per mated male and female are connected through the adult sex ratio (Arnold & Duvall, 1994; Shuster & Wade, 2003). We demonstrated that the observed mean and variance in mate numbers of mated females, the *ASR*, and the predicted ${\overset{¯}{x}}_{m}^{*}$ were inconsistent with a mean and variance of a male mate frequency distribution exhibiting polyandry, which could be possible (given female mate numbers) when the adult sex ratio (*ASR*) = breeding sex ratio (*BSR*), is significantly male‐biased, and all adults mate; or when a male‐biased *BSR* is significantly greater than the *ASR* = 1, resulting from many unmated adult females when females experience strong directional sexual selection. Neither of these conditions were true: The *ASR* was actually female biased, and we did not see evidence of a large fraction of unmated adult females, as with viviparity it is possible to detect mating failure of adult females due to the retention of unfertilized, atrophied broods. Moreover, females do not express any elaborated secondary sexual characters that indicate strong directional sexual selection. Simply put, female mate frequencies are too high to account for males mating once, and males become even more limiting if some do not mate at all—typical in nature. Instead, the data and predicted average number of mates of mated males (${\overset{¯}{x}}_{m}^{*}=2.4$) were consistent with variable male mating success and male mate frequency distributions that fit polygamous mating systems: polygynandry and polyandrogyny. Polygamy is also consistent with weak sexual dimorphism and other male evolutionary responses to receptive females. We illustrate the importance of male polygamy as an evolutionary response to polyandrous females, with the harmonic mean promiscuity of females. Given polyandrous females, sperm mixing, and equal paternity, the average *S. melanops* male is expected to sire about 62% of an average female's brood, but only 25% if his mate was previously sired by three males—far from complete paternity assurance relative to polyandry and monogamy. Consequently, a male must sire additional females and/or increase his paternity share per female to attain greater reproductive success with sperm competition, because his total expected fitness is equal to the product of his number of mates, average female fecundity, and the harmonic mean promiscuity of females (Collet, Richardson, Worley, & Pizzari, 2012; Shuster & Wade, 2003; Wade & Arnold, 1980).

### 4.4 Conservation consequences of divergence in behavior and mating system on the relationship between annual effective population size and reproductive failure

Might differences in behavior and mating systems exert differential impacts on the population growth rates and the recovery of exploited populations of *Sebastes* species? The mating system assessment of *S. melanops* could have broader implications for the conservation of *Sebastes* species, when the effect of nonterritorial polygamy on the relationship between annual (within‐season) *N*_e_ and reproductive failure of adults is compared to this relationship for reproductively territorial congeners. With weak premating sexual selection in a resource‐free, polygamous breeding population, annual *N*_e_ is relatively high before postmating selection, and *N*_e_ is highest for a given level of cumulative reproductive failure of a cohort (Figure 6; Parker & Waite, 1997). In contrast, the *S. inermis* mating habits observed by Shinomiya and Ezaki (1991) revealed that territorial males defend courtship display/mating territories (see also Gingras et al., 1998 for *Sebastes miniatus*). A fuller significance of their study became evident during our evaluations of mating system determinants in the two major life history groups: *S. inermis* meet all the basic physiological and ecological prerequisites for a lekking species (reviewed in Hoglund & Alatalo, 1995). The opportunity for directional sexual selection on males (due to high variance in male mating success, *I*_mates_; Table S3) is expected to rise in territorial species if males are unable to acquire mating territories and are excluded from mating by male–male combat, rejected by female choice of territory size/quality, or when a position effect (for example, the highest area on a rocky reef) is related to a greater female encounter rate (Genner, Young, Haesler, & Joyce, 2008). Another potential source of male fitness variance is from mate copying by females (Dugatkin & Godin, 1993), especially when females aggregate around lekking and territorial males (Wade & Pruett‐Jones, 1990). Taken together, *I*_mates_ is potentially greater in demersal/benthic, territorial *Sebastes* species, when premating sexual selection skews the BSR, increases variation in male reproductive success (as with polygyny), which could reduce the breeding *N*_e_ relative to polygamy in same‐size adult populations of *S. melanops*. Moreover, reproductively territorial species can experience an elevated risk of demographic stochasticity with greater sexual selection (Møller & Legendre, 2001), and be more prone to reproductive failure, annual genetic bottlenecks, and environmental stochasticity. Interestingly, many demersal species that are often solitary as adults and express territorial behavior, are much less common, more patchy in their distributions (and with habitat preferences, e.g., Larson, 1980a,b), and have more harvest restrictions, compared to mid‐water congeners that express schooling, nonterritorial behavior. For some reproductively territorial species, this could be partly attributable to less productive life history correlates of population growth: greater length/age at maturity, greater maximum length/age, etc. (Reynolds, Jennings, & Dulvy, 2001). For others, productivity parameters are similar to schooling, nonterritorial species, and their mating system and behavior could reduce *N*_e_, their per capita birth rates, and subsequent population growth. Clearly, to rebuild rockfish populations, we need more in situ observational studies of reproduction and *N*_e_ estimates for less common territorial species—a considerable challenge. Two conservation objectives, suggested by Rowe and Hutchings (2003), we recommend here, tailored for rockfish. The first is to identify the locations of any mating territories/aggregations, with ROV/SCUBA, and monitor their phenology, sex‐specific movements/migration patterns, and long‐term trends in abundance. The second is to collect sex‐specific biological data on commercial/recreational catch of overfished and uncommon species for stock assessments (including reconstructing and analyzing legacy data).

Our mating system assessment of nonmodel *S. melanops* estimated population parameters from pregnant females, their offspring, and adults in the breeding population to conclude that multiple mating is common in both sexes. Polygamy is also consistent with male evolutionary responses to female receptivity for *S. melanops* and congeners with a mid‐water, schooling, and nonterritorial life history as adults, and with weak expected premating directional sexual selection on males; all support nonterritorial polygamy. Among mating systems, nonterritorial polygamy confers the highest annual *N*_e_ to cohorts, but evaluations of mating system determinants of congeners with a demersal/benthic, reproductively territorial life history revealed they have more possible mechanisms for generating premating sexual selection and a reduced *N*_e_ to cohorts, compared to *S. melanops*. Such a reproductive strategy may be less conducive to population growth and recovery from exploitation.

While the fundamental statistical relationships which could best quantify a mating system (i.e., Bateman Gradients) will likely remain elusive in wild, nonmodel species in the marine realm, our multidisciplinary approach that integrated mating system measures and reproductive life history within the framework of sexual selection theory has revealed an important window into the mating systems and conservation of the *Sebastes* species flock.

## AUTHOR CONTRIBUTION

K. Karageorge contributed to the conception, design, acquisition of data, analysis and interpretation of data, draft revisions, and final approval of the submitted version. R. Wilson contributed to the analysis and interpretation of data, draft revisions, and final approval of the submitted version.

## CONFLICT OF INTERESTS

None declared.
