# Supplementary material for: An integrative mating system assessment of a nonmodel, economically important Pacific rockfish (Sebastes melanops) reveals nonterritorial polygamy and conservation implications for a large species flock
Source: Ecol Evol. 2017 Dec 3;7(24):11277–91. doi: 10.1002/ece3.3579 (PMC5743636; doi:10.1002/ece3.3579)
Supplement: Supplementary file 1 [file ECE3-7-11277-s001.docx]

| **Table S1**. *Sebastes melanops* Parentage Data Determined From Microsatellite Genotypes of Each Mother and Progeny Samples | | | | | | | |
| --- | --- | --- | --- | --- | --- | --- | --- |
| Mother’s ID & Microsatellite Genotypes | Inheritance of maternal alleles in progeny samples in 1:1 ratio ? | | Paternal alleles revealed at each locus from each genotyped mother and progeny sample, n | | Estimated number of sires using single-locus minimum method^2^ | Estimated number of sires using multi-locus data with GERUD2.0 parentage program | Percent of iterations correctly identifying true no. of sires using GERUDsim2.0 |
| DB1^1^  Sal1: 118 or 170/150  Sal3: 123(?)/128  Spi6: ?/126 | Y  Y  Y | | n = 74  118 or 170, 150  123 (?), 128*  102 or 122, 126* | | 1 | 1 | 100% |
| NP9  Sal1: 138/146  Sal3: 118/133  Spi6: 122/122 | Y  Y  Y | | n = 80  142,154  118, 143  122, 138 | | 1 | 1 | 100% |
| NP13  Sal1: 130/142  Sal3: 128/168  Spi6: 134/150 | Y  Y  **N^**^** | | n = 75  130*, 146  128*  122 | | 1 | 1 | 100% |
| NP14  Sal1: 158/166  Sal3: 128/128  Spi6: 114/138 | Y  Y  Y | | n = 70  138  123  126, 134 | | 1 | 1 | 100% |
| Notes: 1. Mother unknown 2. The alleles of the locus with the most paternal alleles were counted, divided by two (for a diploid single sire), and rounded up to the nearest whole number *Allele determined from homozygous progeny; homozygous progeny/paternal allele verified as true, and not null paternal allele and apparent homozygote, by allele segregating with other maternal allele among progeny ** Obs. ratio of maternal alleles in progeny samples significantly different than the expected 1:1 ratio with equal segregation (*P* < 0.05) | | | | | | | |
| **Appendix Table 1**., con’t. | | | | | | | |
| Mother’s ID & Microsatellite Genotypes | Maternal alleles in progeny samples in 1:1 ratio? | | Paternal alleles revealed at each locus from each genotyped mother and progeny sample, n | | Estimated number of sires using single-locus minimum method^2^ | Estimated number of sires using multi-locus data with GERUD2.0 parentage program | Percent of iterations correctly identifying true no. of sires using GERUDsim2.0 |
| NP17  Sal1: 142/142  Sal3: 108/128  Spi6: 114/134 | Y  Y  Y | | n = 73  142*, 162  113, 158  126, 134* | | 1 | 1 | 100% |
| NP18  Sal 1: 154/154  Sal3: 113/128  Spi6: 118/122 | Y  Y  Y | | n = 64  134, 146  128*  118*, 134 | | 1 | 1 | 100% |
| NP15  Sal1: 130/138  Sal3: 113/148  Spi6: 122/138 | Y  Y  Y | | n = 74  138, 166  123, 133, 138  118, 122, 134, 142 | | 2 | 2 | 99.8% |
| DB3  Sal1: 122/138  Sal3: 128/128  Spi6: 130/142 | Y  Y  Y | | n = 72  146, 158, 162  123, 133, 178  118, 122, 126 | | 2 | 2 | 99.6% |
| NP2  Sal1: 130/130  Sal3: 118/138  Spi6: 122/146 | Y  **N^**^**  Y | | n = 82  130*, 134, 138  133, 138*, 148  114, 118, 146* | | 2 | 2 | 99.6% |
| **Appendix Table 1**., con’t. | | | | | | | |
| Mother’s ID & Microsatellite Genotypes | | Maternal alleles in progeny samples in 1:1 ratio? | | Paternal alleles revealed at each locus from each genotyped mother and progeny sample, n | Estimated number of sires using single-locus minimum method^2^ | Estimated number of sires using multi-locus data with GERUD2.0 parentage program | Percent of iterations correctly identifying true no. of sires using GERUDsim2.0 |
| NP16  Sal1: 134/138  Sal3: 128/143  Spi6: 118/138 | Y  Y  Y | | n = 67  122, 126, 130  113, 128*, 133  118* | | 2 | 2 | 99.6% |
| NP21  Sal1: 114/134  Sal3: 113/153  Spi6: 102/118 | | Y  Y  Y | | n = 41  126, 130  108, 113*, 128  102*, 118* | 2 | 2 | 99.5% |
| NP11  Sal1: 130/134  Sal3: 123/128  Spi6: 122/122 | Y  Y  Y | | n = 71  134, 138, 158  118, 133  102, 122 | | 2 | 2 | 99.1% |
| NP10  Sal1: 130/154  Sal3: 113/128  Spi6: 114/118 | Y  Y  Y | | n = 80  130*, 138, 150  118, 128*, 138, 168  102, 130, 146 | | 2 | 3 | 99.0% |
| DB2  Sal1: 126/130  Sal3: 113/123  Spi6: 122/126 | Y  Y  Y | | n = 74  130*, 138, 158, 162  128, 133, 138  122*, 142 | | 2 | 3 | 98.9% |
|  |  | |  | |  |  |  |
| **Appendix Table 1**., con’t. | | | | | | | |
| Mother’s ID & Microsatellite Genotypes | Maternal alleles in progeny samples in 1:1 ratio? | | Paternal alleles revealed at each locus from each genotyped mother and progeny sample, n | | Estimated number of sires using single-locus minimum method^2^ | Estimated number of sires using multi-locus data with GERUD2.0 parentage program | Percent of iterations correctly identifying true no. of sires using GERUDsim2.0 |
| NP6  Sal1: 146/150  Sal3: 128/128  Spi6: 102/126 | Y  Y  Y | | n = 70  130, 134, 138  118, 123, 128*, 138, 153  118, 122, 146 | | 3 | 3 | 98.5% |
| NP7  Sal1: 134/162  Sal3: 128/133  Spi6: 134/162 | Y  Y  Y | | n = 96  130, 142, 146, 154  123, 133, 138  102, 118, 126, 142 | | 2 | 4 | 96.0% |
| DB4  Sal1: 130/138  Sal3: 113/128  Spi6: 102/122 | Y  Y  Y | | n = 93  118, 130*, 138*, 142  118, 128*, 138, 148  102*, 122*, 130, 142, 150 | | 3 | 4 | 95.0% |
