# Supplementary material for: An integrative mating system assessment of a nonmodel, economically important Pacific rockfish (Sebastes melanops) reveals nonterritorial polygamy and conservation implications for a large species flock
Source: Ecol Evol. 2017 Dec 3;7(24):11277–91. doi: 10.1002/ece3.3579 (PMC5743636; doi:10.1002/ece3.3579)
Supplement: Supplementary file 2 [file ECE3-7-11277-s002.docx]

| **Table S2**. Evaluation of Reproductive Life History Traits & Ecology Influencing the Opportunity for Sexual Selection on Males (I_mates_) and the Opportunity for Selection on Females (I_females_) in *Sebastes* species that Express Schooling, Non-Territorial (NT) Behavior, and their Expected Contributions in Shaping the Predicted Sex Difference in Opportunity for Selection, ∆I | |
| --- | --- |
| Influence of female spatiotemporal distribution in receptivity^1^ on the opportunity for sex. sel. on males, I_mates_^2,3^: | Mean spatial crowding = m*, & mean temporal crowding = t*, of receptive ♀’s^3^ (low, moderate, high): m*= moderate for ♀’s in NT spp., indicated by ♂ *S. mystinus* search behavior ^3,4^; t*= moderate/high, indicated by OSR of courting/mating groups near conspecific schools^4,5^, lower for spp. with longer reproductive life spans (population-level asynchrony in ♀ receptivity due to age variability) |
| Influence of female reproductive life history traits on within-female relative fitness variance components contributing to the opportunity for selection on clutch number, I_clutch_: | I_cs, sires_ = effects of different sires (♂ quality) on clutch size^3^ > 0; ♂ seminal contributions may enhance fecundity^6^; genetic incompatibility likely due to significant embryo failure^7^ in *Sebastes*^8,9,10^; polyandrous ♀’s sampling sperm/alleles from multiple mates (Fig. 4b) may enhance offspring viability, ♀ fitness with “compatible genes”^11,12^ |
|  | I_cs, clutch_= effects of variation in clutch numbers/iteroparity on clutch size^3^: I_cs, clutch_ = I_cs, sires_ for *S. melanops* & multiseasonal uniparous spp. due to sub-clutches with different sires (Fig. 3); > 0 for multiseasonal multiparous *Sebastes* spp. with 1 to 3 clutches per year^13^, I_mates_ reduced if each clutch sired by different ♂^3^ |
| I_females_: total oppor. for selection on female fitness (offspring numbers)^2,3^: | >> 0; large *S. melanops* ♀’s may have nearly 4 times the offspring of small ♀’s, high variation in offspring numbers within a female length-class^10^; mating success increases reproductive success if this relationship, "Bateman gradient", is positive^6,14^ |
| Mean & variance in the number of sires of mated female *S. melanops*^†^: | $\bar{x}_{f}^{*}$= 2 & $v_{f}^{*}$= 1, mean smaller & variance larger if some ♀’s fail to mate^15^; parameters affected by operational & breeding sex ratios^6,14,16^, mate quality (e.g., male size)^17^, intensity of sexual selection^6,14^, & geographic variation^18^ |
| Predicted sex difference in opportunity for selection, ∆I = I_males_ - I_females_ ≈ I_mates_ - I_clutch_ (opportunity for sexual selection)^3,19^: | I_males_ ≥ I_females_; I_males_ reduced by polyandrous ♀’s, prolonged ♀ receptivity, moderate or high m*, synchrony in ♀ receptivity allows more ♂’s to mate, I_females_ large; also supported by constrained evol. responses: limited alt. mating phenotypes, weak sex. dimorphism (see Table 1) |
| Sources: 1. Emlen & Oring (1977); 2. Wade & Arnold (1980); 3. Shuster & Wade (2003); 4. Helvey (1982); 5. *S. emphaeus*: W. Palsson, pers. comm.., NOAA Fisheries; 6. Arnold & Duvall (1994); 7. Zeh & Zeh (1996); 8. Boehlert et al. (1982); 9. Boehlert et al. (1986); 10. Bobko & Berkeley (2004); 11. Newcomer et al. (1999); 12. Simmons (2005); 13. Love et al. (1990); 14. Jones et al. (2000a); 15. Shuster (2009); 16. Jones et al. (2004); 17. Jones et al. (2000b); 18. Trexler et al. (1997); 19. Wade (1987)  † Estimated in this study | |

| **Table S3**. Evaluation of Reproductive Life History Traits & Ecology Influencing the Opportunity for Sexual Selection on Males (I_mates_) and the Opportunity for Selection on Females (I_females_) in Benthic/Demersal *Sebastes* species that Express Territorial Behavior (T), and their Expected Contributions in Shaping the Predicted Sex Difference in Opportunity for Selection, ∆I | |
| --- | --- |
| Influence of female spatiotemporal distribution in receptivity^1^ on the opportunity for sex. sel. on males, I_mates_ ^2,3^: | Mean spatial crowding = m* & mean temporal crowding = t* of receptive ♀’s^3^ (low, moderate, high): m*= high for ♀ *S. inermis* & TT spp. aggregated around ♂ territories^4,5^; t*= low /moderate, lower for spp. with longer reproductive life spans (population-level asynchrony in ♀ receptivity due to age variability), prolonged ♀ receptivity allows more ♂’s to mate^3^ |
| Influence of female reproductive life history traits on within-female relative fitness variance components contributing to the opportunity for selection on clutch number, I_clutch_: | I_cs, sires_ > 0 if broods multiply sired |
|  | I_cs, clutch_= I_cs, sires_ for territorial *Sebastes* spp with sub-clutches from different sires |
| I_females_: total oppor. for selection on female fitness (offspring numbers)^2,3^: | >> 0; larger in territorial *Sebastes* spp. that have later age & length at maturation, larger body size & longer lifespans than in non-territorial spp (i.e., life history parameters L_mat_, T_mat_, L_∞_, & T_max_)^6^ |
| Mean & variance in the number of sires of mated females in territorial *Sebastes* spp. | Unknown, but polygamy observed in apparent lekking *S. inermis*^4^ |
| Predicted sex difference in opportunity for selection, ∆I = I_males_ - I_females_ ≈ I_mates_ - I_clutch_ (opportunity for sexual selection)^3,7^: | I_males_ > I_females_; I_mates_ reduced by polyandrous ♀’s and prolonged ♀ receptivity, I_females_ large; ∆I larger for TT *Sebastes* if ♂’s without mating territories fail to mate &/or mate copying^8^ by aggregating ♀’s^9^ |
| Sources: 1. Emlen & Oring (1977); 2. Wade & Arnold (1980); 3. Shuster & Wade (2003); 4. Shinomiya & Ezaki (1982); 5. *S. miniatus*: M. Gingras, pers. comm., California Dept. Fish & Wildlife; 6. Love et al. (1990); 7. Wade (1987); 8. Dugatkin & Godin (1993); 9. Wade & Pruett-Jones (1990)  † Estimated in this study | |
