# Supplementary material for: An integrative mating system assessment of a nonmodel, economically important Pacific rockfish (Sebastes melanops) reveals nonterritorial polygamy and conservation implications for a large species flock
Source: Ecol Evol. 2017 Dec 3;7(24):11277–91. doi: 10.1002/ece3.3579 (PMC5743636; doi:10.1002/ece3.3579)
Supplement: Supplementary file 3 [file ECE3-7-11277-s003.docx]

| Effect of unmated adult males, due to pre-mating sexual selection (male-male competition and/or female choice), on total mean male mating success, $\bar{x}_{m}$, relative to $\bar{x}_{m}^{*}$ | | | | |  |  |  |  |
| --- | --- | --- | --- | --- | --- | --- | --- | --- |
| 0.6 | 0.8 | 1.0 | **1.2** | 1.4 |  |  |  |  |
| ${\bar{\boldsymbol{x}}}_{\mathbf{f}}^{\mathbf{*}}$ | 2.5 | 1.5 | 2.0 | 2.5 | 3.0 | 3.5 | 2.5 |  |
|  | **2.0** | 1.2 | 1.6 | 2.0 | **2.4** | 2.8 | **2.0** |  |
|  | 1.5 | 0.9 | 1.2 | 1.5 | 1.8 | 2.1 | 1.5 |  |
|  | 0.6 | 0.8 | 1.0 | **1.2** | 1.4 |  | |  |
| $ASR= n_{\mathrm{adult}♀}/n_{\mathrm{adult}♂}$  Effect of underestimating adult males on the adult sex ratio sample, *ASR*, and on the estimated mean number of mates of mated males, $\bar{x}_{m}^{*}$ (matrix cells) | | | | |  |  |  |  |

**Figure S1.** Quantifying the effects of potential errors in the estimated average number of sires per mated female ($\bar{x}_{f}^{*}$, corresponding to the rows of the matrix) and the estimated adult sex ratio (*ASR*, corresponding to the columns of the matrix), on the estimated average number of mates per mated male ($\bar{x}_{m}^{*}$, values inside matrix). Values estimated from the data are indicated in bold. The mean number of mates of mated males, $\bar{x}_{m}^{*}$= 2.4, was estimated from the product of the mean number of mates of mated females, $\bar{x}_{f}^{*}$= 2.0, determined from parentage results of the brooded females, and the *ASR* = 1.2. Effects of sampling error and unmated males that could upwardly bias the estimate of$\bar{x}_{m}^{*}$, are indicated. Shaded adult sex ratio values are statistically indistinguishable from 1.

**Appendix S1**

***Evaluation of the influence of male and female reproductive life history and ecology on shaping the sex difference in opportunity for selection*.—**We investigated the underlying causal sexual selection mechanisms that can shape the evolutionary responses of males and the overall mating systems of *S. melanops* and congeners, grouped by their two major life history patterns. We evaluated how male and female reproductive life history traits and the environment are expected to influence their relative fitness variances and the magnitude of the sex difference in opportunity for selection. Our mating system inference of polygamy and the observations of evolutionary responses of males were evaluated against the general theoretical predictions based on sexual selection theory, to address the questions: Is polygamy consistent with the expected sex difference in the strength of selection? Why do the sexes appear remarkably similar in appearance? Is the intensity of sexual selection potentially greater in *Sebastes* species with different mating systems and life history patterns, and if so, what are the sexual selection mechanisms responsible and the implications for their conservation?

The sex difference in opportunity for sexual selection identifies the direction and degree to which sexual dimorphism is expected to evolve (Shuster, 2009; Shuster and Wade, 2003; Wade, 1987)_,_ and is a predictor of the potential intensity and direction of sexual selection (a surrogate for sex-specific relationships between reproductive and mating success = Bateman gradients). The opportunity for sexual selection (I_mates_ for males) and the opportunity for selection (I_males_ and I_females_) are the variances in mating success and reproductive success, respectively, divided by the squares of their means, and represent the upper limits on the strength of sexual selection, selection on offspring production, and the rate of evolutionary change in mean fitness acting on a population (Arnold and Wade, 1984b; Arnold and Wade, 1984a; Crow, 1958; Crow, 1962). I_males_ and I_females_ are related through the sex ratio, and if one, the opportunity for sexual selection is approximately equal to the sex difference in opportunity for selection I_males_ – I_females_ = I_mates_, and is largely dependent upon the variance in male mating success when females are monogamous and semelparous (Wade, 1979; Wade and Arnold, 1980). Wade (1987) showed polyandrous mating and iteroparity to be similar in their effects on I_females_, as with multiple mating by males, because each clutch is divided into sub-clutches, equal in number to the number of sires (see also Wade and Arnold, 1980). I_clutch_ describes the variance in clutch number among females, V_clutch_ divided by the squared average number of clutches per female (C^2^). Therefore, I_males_ = (I_females_- I _clutch_) + I _mates_, and hence ∆I = I_males_ – I_females_ ≈ I_mates_ - I_clutch_, and the ∆I is increased by variation in mating success among males (I_mates_), and is reduced by variation in mating and reproductive success among females (I_clutch_). I_clutch_ is also influenced by components of variance in reproductive success among females that effect variation in female clutch size (cs): V_cs,sires_, the sires within-female component of variance in offspring numbers due to the effects of mating with different sires (i.e., male quality); and V_cs,clutch_, the within-female component of variance in offspring numbers due to the effects of different numbers of clutches per female (Shuster et al., 2013; Shuster and Wade, 2003). Divided by the squared average of female offspring numbers, I_cs, sires_ and I_cs,clutch_ identify how individual female fitness (cs) becomes variable from the affects of female life history when classified by mate numbers (monogamous or polyandrous) and reproductive episodes (semelparous or iteroparous), and we identify their expected effects on I_females_ and ∆I. We examined the mean spatial and temporal crowding of receptive females (Shuster and Wade, 2003) influencing I_mates_ in TT and NT *Sebastes* (broadly categorized as low, moderate, or high), because male monopolization of receptive females by mate guarding, defense of mating territories, and control of environmental resources used by females can produce high variance in male mating success (Emlen and Oring, 1977). The ∆I was evaluated based on the expected size of I_mates_ relative to I_clutch_.

***Evaluation Results.—***The sex difference in opportunity for selection, ∆I (= I_males_ - I_females_ ≈ I_mates_ - I_clutch_), i.e., the opportunity for sexual selection, is expected to be small (I_males_ ≥ I_females_ ≈ I_mates_ ≥ I_clutch_) for *S. melanops*, primarily due to the absence of male mating territories and associated sexual selection mechanisms that constrain I_mates_ , relative to polyandrous mating that increases I_clutch_ (Appendix Table 2). The ∆I is potentially greater in TT *Sebastes* species due to male-male competition for mating territories, female mate choice of male territories/dominant males, and a strong potential for mate copying by females aggregating around male territories, including leks (Appendix Table 3).

***Discussion*.—**The influence of male and female reproductive life history and ecology on shaping the sex difference in opportunity for selection (∆I = I_males_ - I_females_ ≈ I_mates_ - I_clutch_), or the opportunity for sexual selection, is in agreement with the evolutionary responses of schooling, non-territorial *Sebastes* species, and a non-territorial polygamy *S. melanops* mating system (Appendix Table 2). The ∆I is expected to be diminished by the opportunity for selection on female clutch number, I_clutch_, because the production of multiple sub-clutches (each with a different male) from polyandrous mating, interchangeably diminishes the variance in fitness among males (I_mates_) by enabling more males to mate, and adds to the variance in fitness among females (Collet et al., 2012; Shuster et al., 2013; Shuster and Wade, 2003). Stated differently, the number of mates is a potentially major source of fitness variance for both males and females, if the least-squares regressions of reproductive success on mating success, i.e., the sexual selection or Bateman gradients (after Bateman, 1948), are positive (Arnold and Duvall, 1994; Jones et al., 2000a). Within polyandrous females, the variance in offspring numbers is increased in part by the sires-within-females variance in relative fitness component, I_cs, sires_ > 0, due to the effects of mating with different sires, i.e. male quality (Shuster and Wade, 2003). Rigorous fecundity estimates conducted on *S. melanops* by Bobko and Berkeley (2004) found that post-fertilization fecundity was highly variable for a given female length, and the number of mates/sub-clutches (I_clutch_) and male quality (I_cs,sires_) are candidate sources of this variation, due to multiple paternity and genetic incompatibility.

Male fitness variance (I_mates_) is constrained in schooling, non-territorial *Sebastes* species that do not directly utilize environmental resources for reproduction, eliminating male-male competition for mating territories (intrasexual selection), and dominant-male monopolization of females that may otherwise have strong preferences for those males/resources (intersexual selection; though female choice for larger/older males is expected). This, in addition to polyandrous females, expected prolonged female receptivity, females with a moderate spatiotemporal distribution in their receptivity, and adult populations with an even *ASR* all facilitate more mating males and erode the opportunity for directional sexual selection on males. Thus, the requirement for sex differences in phenotypes (sexual dimorphism), opposing directional selection between phenotypic trait means, is diminished by a small expected ∆I (Shuster and Wade, 2003; Wade, 1987) conveyed by *S. melanops* (i.e., I_males_ ≥ I_females_ ≈ I_mates_ ≥ I_clutch_) that express weak sexual dimorphism (transient courtship displays are expected to be most prominent secondary sexual trait, as with other *Sebastes species*). A small expected ∆I also corresponds with polygamy, because the sexes are expected to experience a similar strength of sexual selection (and Bateman gradients), though stronger on males that invest less in offspring (Arnold & Duvall 1994).
